# Inpatient medical management of severe pediatric obesity: Literature review and case reports

**DOI:** 10.3389/fped.2023.1095144

**Published:** 2023-02-13

**Authors:** Victoria E. Goldman, Juan C. Espinoza, Alaina P. Vidmar

**Affiliations:** ^1^ Department of Pediatrics, Children's Hospital Los Angeles, Los Angeles, CA, United States; ^2^ Keck School of Medicine of University of Southern California, Los Angeles, CA, United States; ^3^ Department of Pediatrics, Endocrinology and Metabolism, Children's Hospital Los Angeles Center for Diabetes, Los Angeles, CA, United States

**Keywords:** case report, weight management, inpatient hospitalization, obesity, weight loss

## Abstract

Pediatric obesity rates continue to rise steeply with significant adverse effects on health outcomes across the lifespan. Significant obesity can affect the efficacy, side effects, and ability to use certain treatment, medication, or imaging modalities needed in the evaluation and management of acute pediatric conditions. Inpatient settings are rarely used as an opportunity for weight counseling and thus there is a paucity of clinical guidelines on how to manage severe obesity in the inpatient setting. We present a literature review and three patient cases with single-center protocol for non-surgical management of severe obesity in children admitted for other acute medical reasons. We performed a PubMed review from January 2002 to February 2022 utilizing keywords: “inpatient,” “obesity,” and “intervention.” For our cases, we identified three patients with severe obesity acutely impacting their health while admitted for medical treatment who concurrently underwent acute, inpatient, weight loss regimens at a single children's hospital. The literature search yielded 33 articles describing inpatient weight loss treatments. Three patients met case criteria, all three of which demonstrated a decrease in their weight in excess percent of the 95th percentile after inpatient weight-management protocol implementation (% reduction BMI_p95_: 16%–30%). This highlights obesity acutely limits or impacts specific medical care required during inpatient admissions in pediatric patients. It also suggests that implementation of an inpatient weight-management protocol during admission may provide an opportune setting to support acute weight loss and overall improved health outcomes in this high-risk cohort.

## Introduction

One in five children (ages 2–19 years) has obesity in the United States. Pediatric obesity is considered one of the most serious global health problems of this century, with rates projected to increase 130% over the next two decades (1, 2). Hospitals are seeing increased rates of severe pediatric obesity impacting treatment and outcomes for acute hospitalizations, regardless of presenting diagnosis (1, 3–6). Inpatient hospitalization presents an opportunity to engage in nutritional counseling, obesity-related comorbidity screening, and initiation of anti-obesity pharmacotherapy (7, 8). This is similar to screening and counseling for other conditions while inpatient, yet studies show these assessments rarely occur (7, 9, 10). There is minimal data about inpatient obesity interventions, despite the fact that severe obesity affects the acute needs, treatment, and outcomes for many medical conditions in both adult and pediatric cohorts (11). This absence of information presents major challenges to pediatric healthcare systems attempting to provide long-term, comprehensive care for youth with obesity.

Pediatric obesity independently increases morbidity and mortality across the lifespan (4, 5). Obesity has been linked to worse outcomes in hospitalized patients with prolonged length of stay, greater costs, increased infections, and increased post-operative complications (4, 5, 12–14). In pediatrics, specifically, there are significant medication safety risks for youth living with obesity, given that the majority of medications utilized inpatient have weight-based dosing. In fact, three recent reviews found that two-thirds of all prescribed medications in children with obesity were either sub-therapeutic or supra-therapeutic (1, 12, 15).

Despite negative inpatient outcomes, studies show that obesity is rarely documented or considered an important factor in inpatient care, unless it is thought to directly affect the treatment for the acute illness (6, 8). A recent study found that fewer than 40% of children's hospitals have universal policies to identify and treat patients with obesity, with BMI calculated in only 35% of pediatric inpatient encounters (12). This is particularly problematic as adult studies have found that the only factor associated with increased likelihood of treatment for obesity while inpatient was a diagnosis of obesity in the medical record (7). A pediatric study found that a documented diagnosis of obesity was associated with being 35 times more likely to receive interventions while hospitalized (9). Although there is a paucity of literature exploring weight loss counseling during acute hospitalizations, data collected in adults with obesity admitted for non-weight related conditions revealed that the majority of patients with obesity are receptive to weight loss advice while hospitalized (16).

Weight loss, even small percentages, can have long-lasting effects on health, particularly in children. BMI *z*-score decrease of 0.5 standard deviations has been positively correlated with improved cardiovascular risk profiles and improved lung function (17, 18). Weight loss, in the acute setting, has also been shown to have immediate positive outcomes. This is best exemplified in bariatric surgery literature, which demonstrates pre-operative weight loss is correlated with reduced operative time, blood loss, complications, liver size, and length of stay, and greater weight loss following surgery (19, 20). There is growing evidence that inpatient weight-management programs are more successful at achieving acute weight loss than outpatient treatments (17, 21, 22). In a 2011, 22-study systematic review, there was a 191% greater reduction in individuals with obesity in pediatric inpatient weight loss programs as compared to outpatient (23). In fact, even short-term inpatient weight loss treatments have been shown to be more effective than long-term outpatient intervention (24). Unfortunately, there is little data on how to incorporate weight-management strategies into inpatient encounters in youth with obesity (19).

Given research and treatment gaps for pediatric inpatient obesity management, we conducted a review of inpatient weight-management treatment in both children and adults. The objective of this article is to describe the existing literature on inpatient weight loss treatments and present examples of acute weight loss in hospitalized pediatric patients that may serve as models for future inpatient weight loss interventions. We report three cases of pediatric patients, with severe obesity affecting their immediate hospitalization, and the weight-management treatment they underwent at a single, urban, quaternary care, children's hospital.

## Methods

A search was conducted through the PubMed database focused on non-surgical, inpatient, obesity weight loss treatments. The key words included: “inpatient,” “obesity,” and “intervention.” The search terms were intentionally broad to capture relevant studies while preventing omissions. The search was conducted in March 2022 and included date limits of January 2002 through February 2022. Results were screened to include articles reporting on inpatient weight loss treatments for patients with obesity. The search was not narrowed to pediatric patient populations due to the limited number of articles. Initial search of articles was conducted manually by one reviewer (VG). The database search resulted in an initial pool of 208 articles. Articles in languages other than English, and articles outside the defined time period were removed. All records were then independently reviewed by two reviewers (VG and AV) using the inclusion criteria. Given the relatively small number of search results and nuanced subject focus, all articles were evaluated for inclusion rather than having a separate screening phase. Articles that focused primarily on surgical weight loss treatment for obesity were excluded. Discrepancies were resolved through discussion between investigators. Data foci included: date, country, study design, population, duration, primary outcome(s) with results, and weight loss.

Several patient cases of pediatric, inpatient, weight loss from an urban, freestanding children's hospital caring for diverse communities were closely examined and three cases are presented as examples of this intervention. All presented cases include pediatric patients with severe obesity (120% of the 95th percentile for body mass index) for whom acute weight loss was recommended to either improve current health or better prepare for a specific therapy or treatment.

## Results

**Figure 1:**
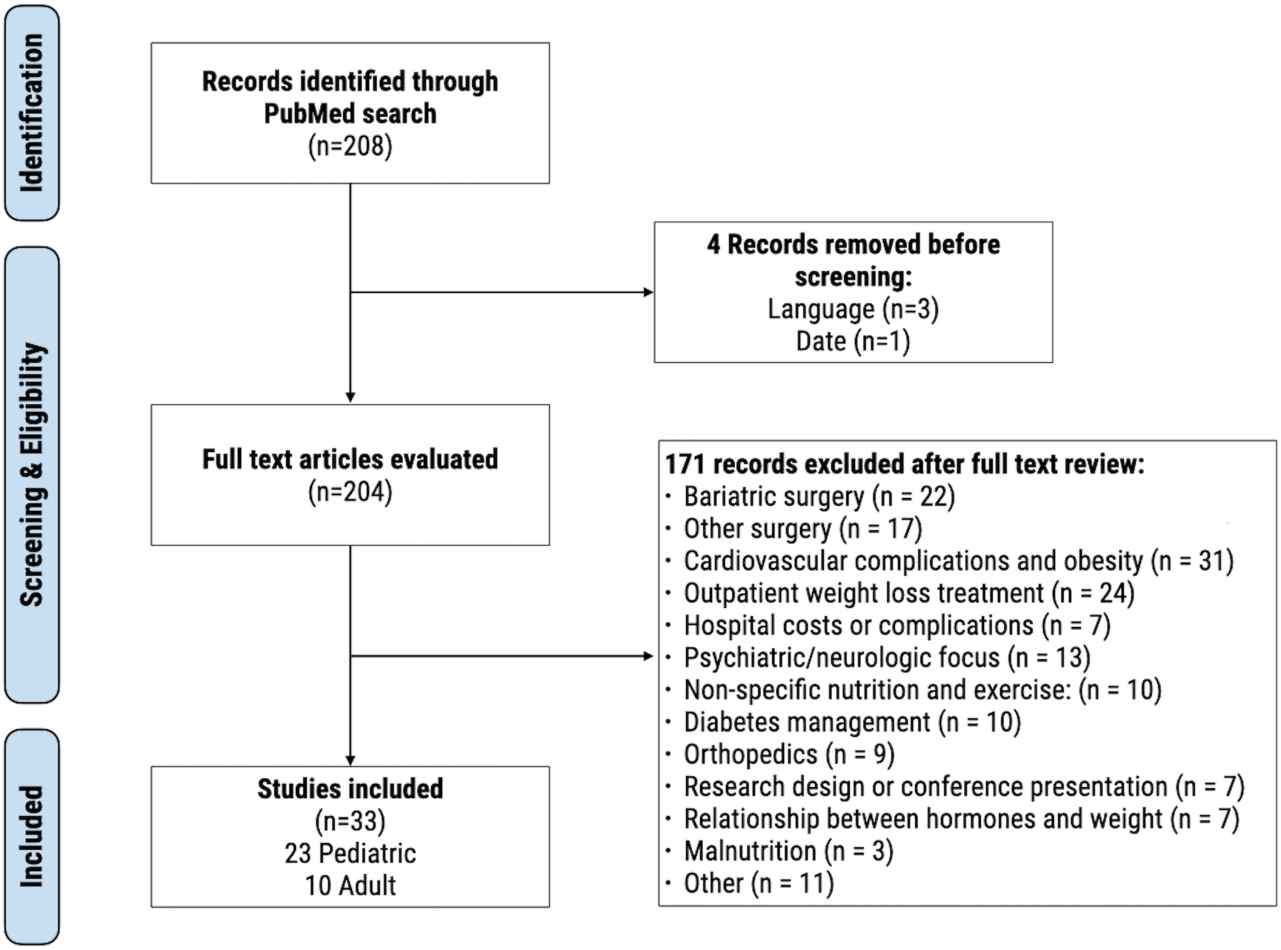
Modified PRISMA flow diagram for our literature search results on inpatient, non-surgical, weight loss interventions for adult and pediatric patients.

The review examining inpatient weight loss treatments for patients with obesity found 208 results (Figure 1). Four articles were removed for language (*n* = 3) and publication date (*n* = 1). The remaining 204 articles were screened and 171 were secondarily excluded due to wrong primary foci including: bariatric surgery (*n* = 22), other surgery (*n* = 17), cardiovascular complications (*n* = 31), outpatient weight loss (*n* = 24), hospital costs or complications (*n* = 7), psychiatric/neurologic focus related to obesity (*n* = 13), non-specific nutrition and exercise: (*n* = 10), research design or conference presentation (*n* = 7), diabetes (*n* = 10), orthopedics (*n* = 9), hormones and weight association (*n* = 7), malnutrition (*n* = 3), and other (*n* = 11). 33 studies met full criteria, including 23 pediatric and 10 adult studies. Sample sizes ranged from 1 to 1,862 individuals, with four being the youngest age included. Length of interventions ranged from 7 days to 10 months. Publications included 26 clinical trials, 2 case reports, 2 review articles, 1 prospective intervention study, 1 comparative study, and 1 case series. All studies included males and females, except for 1 adult study and single-patient case reports. Results focused on weight loss, psychologic impacts of weight, quality of life, cognitive functioning, eating behaviors, and biochemical parameters associated with weight loss.

Few studies discussed the longevity of the interventions more than one year after the interventions or the recidivism rates. One study examined 3 and 5 year follow ups and demonstrated continued reduction of BMI-SDS (by 0.20 and 0.15 respectively) as well as health related behaviors (24).

### Pediatric literature review

Among the 23 pediatric studies included (Table 1), the ages ranged from 4 to 19 years. There were 18 clinical trials, 2 case reports, 1 prospective intervention study, 1 comparative study, and 1 case series. All studies showed decreased weight in a portion of participants, as indicated by BMI, weight, BMI SDS, fat mass, and other measures. In addition, 5 studies also reported improved quality of life, 3 reported increased athletic fitness (i.e., athletic competence, aerobic fitness), 6 improved behavioral factors, 2 improved cognitive functioning, and 6 improved biochemical parameters.

**Table 1:** Characteristics of pediatric studies meeting inclusion criteria.

Article	Date	Country	Study Design	Intervention	Population	Length	Primary Outcome (s)	Result	Weight Loss
Warschburger et al. (25)	2001	Germany	Controlled Clinical Trial	Cognitive-behavioral training (CBT) program focused on obesity and psychosocial consequences	197 individuals *(vs. 76 controls)* - Ages 9–19 - Number female, male unspecified	6 weeks	1. Weight loss 2. Decreased psychological distress associated with obesity	1. Decreased weight 2. Improved quality of life, eating behaviors	15% reduction in obesity prevalence in intervention group (compared to 10% in control)
Knöpfli et al. (21)	2008	Switzerland	Clinical Trial	Multidisciplinary inpatient obesity program with nutritional, physical activity, behavioral components	130 individuals - Ages 4–19 - 52 female, 78 male *Exclusion: secondary obesity, endocrinologic disease*	8 weeks	1. Weight loss	1. Decreased weight 2. Increased aerobic fitness and quality of life	12.7 kg weight decrease
Messersmith et al. (26)	2008	USA	Case Report	Interdisciplinary behavioral intervention complementing nutritional, physical therapy, exercise regimen	1 individual *with Prader-Willi Syndrome, pulmonary hypertension, heart failure* - Age 15	5 months	1. Decreased BMI	1. Decreased BMI 2. Decreased tantrums	24.5 kg/m^2^ BMI decrease
Adam et al. (27)	2009	Germany	Controlled Clinical Trial	Interdisciplinary intervention with diet/nutritional education, exercise, behavioral therapy	162 individuals *(vs. 75 controls)* - Ages 10–15 - 94 female, 68 male	6 weeks *+10.5 months outpatient*	1. Weight loss	1. Decreased weight *(BMI decrease ≥ 0.2)* in 65.5% 2. Improved eating behavior, quality of life	0.36 BMI SDS decrease *Vs. 0.04 control*
Aerberli et al. (28)	2010	Switzerland	Clinical Trial	Multidisciplinary program with caloric restriction, physical activity, behavioral modification	206 individuals - Ages 10–18 - 87 female, 119 male *Exclusion: secondary obesity, endocrinologic disease, major medical problems*	2 months	1. Predictive value of TSH for insulin sensitivity (independent of weight loss) 2. Weight loss	1. Decreased TSH predictive of decreases in fasting insulin 2. Decreased weight	14.4 kg weight decrease 0.42 BMI SDS decrease
Murer et al. (29)	2011	Switzerland	Clinical Trial	Multidisciplinary program with caloric restriction, physical activity, behavioral modification	203 individuals - Ages <18 - 89 female, 114 male *Exclusion: secondary obesity, endocrinologic disease, other major medical problems*	2 months	1. Predictive value of leptin on metabolic variables 2. Weight loss	1. 76% leptin decrease, improved glucose + lipid metabolism 2. Decreased weight	13.9 kg weight decrease
Adam et al. (24)	2013	Germany	Clinical Trial	Interdisciplinary intervention with diet/nutritional education, exercise, behavioral therapy	604 individuals - Ages 10–15 - 350 female, 254 male	6 weeks *+ 10.5 months outpatient*	1. Weight loss	1. Decreased weight, maintained at 5 years 2. Increased quality of life (greatest change inpatient)	0.275 BMI SDS decrease
Karner-Rezek et al. (30)	2013	Switzerland	Clinical Trial	Multicomponent program with goal energy deficit of 500 kcal through nutritional and physical activity interventions	28 individuals - Ages <18 - 9 female, 19 male *Exclusion: secondary obesity, underlying disease*	8 weeks	1. Impact of high intensity exercise on body mass and composition; correlation with baseline anaerobic fitness	1. Decreased fat mass 2. Weight loss	11 kg/m^2^ BMI decrease
Kruger et al. (19)	2013	USA	Case Report	Pre-operative program with high-protein, sugar-free liquid diet and physical activity plan	1 individual - Age 16 - Male	13 days	1. Weight loss prior to bariatric surgery 2. Feasibility of intervention	1. Decreased weight 2. Successful intervention	13 kg inpatient weight decrease *22 kg preoperatively*
Siegrist et al. (31)	2013	Germany	Clinical Trial	Interdisciplinary program with physical activity, diet, behavioral therapy	402 individuals - Ages 6–19 - 238 female, 164 male *Exclusion: syndromal obesity, endocrinologic disease*	4–6 weeks	1. Weight loss 2. Correlation of weight loss with adiponectin / leptin	1. Decreased weight 2. Baseline BMI positively associated with Leptin 3. Neither BMI nor leptin predicted long-term weight loss	3.3 kg/m^2^ BMI decrease
Verbeken et al. (32)	2013	Belgium	Randomized Controlled Trial	Executive Functioning training with game elements on weight loss maintenance in addition to non-diet healthy lifestyle program	44 individuals - Ages 8–14 years - 20 female, 24 male	10 months *6 weeks executive functioning training vs. control*	1. Weight loss 2. Effect of executive functioning training with game elements on weight loss maintenance	1. Decreased weight 2. Improved working memory 3. Better weight loss maintenance in intervention group	5.29 kg/m^2^ BMI decrease *Vs. 5.22 kg/m^2^ BMI decrease - control* *12 week follow up: −0.50 kg/m^2^ vs. + 1.2 kg/m^2^ in control*
Van der Baan-Slootweg et al. (17)	2014	Netherlands	Randomized Clinical Trial	Intensive, family-based lifestyle intervention with exercise, nutritional education, behavior modification (for patient and caregiver)	90 individuals - Ages 8–18 - 52 female; 38 male	6 months	1. Change in BMI *z*-score 2. Comparison of inpatient vs. ambulatory BMI *z*-score	1. Decreased BMI *z*-score 2. Greater decrease in inpatient group 3. Decreased fasting insulin, total cholesterol, LDL, triglycerides, fat mass, Vo_2_	0.61 BMI SDS decrease *Vs. 0.35 BMI SDS decrease - ambulatory*
Vrablik et al. (33)	2014	Czech Republic	Clinical Trial	Intensive lifestyle intervention with individualized dietary changes and exercise	309 individuals - Ages 8–15 - 194 female, 115 male	1 month	1. Weight loss 2. Effect of lifestyle intervention on biomarkers of cardiometabolic risk	1. Decreased weight 2. Decreased biochemical parameters (triglycerides, cholesterol, LDL, HDL, apoB, insulin, c-peptide)	6.4 kg weight decrease 2.35 kg/m^2^ BMI decrease
Kokkvoll et al. (34)	2015	Norway	Randomized Control Trial	Multiple-family intervention with multidisciplinary team focused on group-based physical activity, counseling, nutritional education	97 individuals - Ages 6–12 - 49 female, 42 male	7 days	1. Weight loss 2. Comparison of multiple-family, partially inpatient, weekly activities vs. single family intervention with counseling	1. BMI decreased in both groups, greater with inpatient intervention 2. Improved athletic competence, social acceptance, behavioral conduct	0.20 BMI SDS decrease *Vs. 0.08 BMI SDS decrease non-inpatient*
Halberstadt et al. (35)	2016	Netherlands	Prospective Intervention Study	Intensive, multidisciplinary lifestyle intervention focused on eating styles	120 individuals - Ages 8–19 - 81 female, 39 male	2–6 months *12 months total*	1. Weight loss 2. Association of eating style with weight loss	1. Decreased weight 2. Self-motivation positive protective factor; previous dieting: negative predictive factor	4.29 kg/m^2^ BMI decrease 0.41BMI SDS decrease
Koot et al. (36)	2016	Netherlands	Comparative Study	Intensive lifestyle treatment with nutrition and behavior modification for patients and caregivers, exercise	51 individuals -18 female, 33 male *Exclusion: No non-alcoholic fatty liver disease*	2–6 months	1. Change in liver steatosis 2. Weight loss	1. 43% normalization liver fat content 43% 2. Weight decreased 3. ALT normalization	0.37 kg/m^2^ BMI decrease *Vs. 0.16 kg/m^2^ decrease outpatient*
Schiel et al. (37)	2016	Germany	Clinical Trial	Structured Treatment and Teaching Program (STTP) with 28 therapeutic sessions (i.e. psychosocial, nutrition, physical activity)	143 individuals - Ages 9–18 - 89 female; 54 male	6 weeks	1. Weight loss	1. Decreased weight 2. Most important factors for weight reduction: resilience, absence of intrafamilial conflict, structured daily schedule	5.52 kg decrease
Taylor et al. (38)	2016	United States	Case Series	Comprehensive multidisciplinary intervention with adolescent medicine specialists, dieticians, psychologists, etc.	3 individuals *(2 inpatient)* - Ages 15–16 - 1 female, 2 male	2–3 months	1. Weight loss	1. Weight decreased in 1 inpatient, weight gain in 1 inpatient	Inpatient Decrease: 140.7 kg, 25.77 kg/m^2^ Inpatient Gain: 118.8 kg, 13.4 kg/m^2^
Warschburger et al. (39)	2016	Germany	Randomized Controlled Trial	Multidisciplinary inpatient rehabilitation with diet modification, activity sessions, CBT, behaviorally oriented parent training program	686 individuals - Ages 7–13	3–6 weeks	1. Weight loss 2. Parent skills *Cognitive behavioral group sessions vs. written information*	1. Decreased weight 2. No difference between groups 3. Increased quality of life, healthy food intake, exercise	0.24 BMI SDS decrease
Vantieghem et al. (40)	2018	Belgium	Controlled Clinical Trial	Multidisciplinary obesity treatment program with exercise program, dietary changes	62 individuals - Ages 12–18 - 44 female; 18 male	30 weeks	1. Weight loss 2. Cognitive functioning	1. Weight decreased 2. Improved attention; short term memory uncorrelated with weight loss	19.06 kg decrease 7.69 kg/m^2^ BMI decrease
Miguet et al. (41)	2020	France	Clinical Trial	Multidisciplinary weight loss program with physical activity, nutritional education, psychological support	24 individuals - Ages: 11–15 - 23 female, 7 male *Exclusion: specific medications, more than 2 h physical activity/week*	10 months	1. Weight loss 2. Impact on food reward	1. Weight decreased 2. Improved: decreased appetite, emotional/ uncontrolled eating	8.9 kg decrease
Thivel et al. (42)	2020	France	Randomized Control Trial	Inpatient multidisciplinary weight-loss intervention with eccentric or concentric cycling	24 individuals - Ages: 12–16 - 12 female, 12 male *Exclusion criteria: regular tobacco/alcohol use, specific medications*	12 weeks	1. Weight loss 2. Assessment of eccentric training in weight loss	1. Decreased BMI, fat mass 2. Greater decrease in fat mass in eccentric vs. concentric training	5.0 kg/m^2^ average BMI decrease *5.8 kg/m^2^ eccentric vs. 4.2 kg/m^2^ concentric*
Khammassi et al. (43)	2021	France	Clinical Trial	Multidisciplinary weight-management program with physical activity, nutritional education, psychological support	92 individuals - Ages 12–15 - 62 female, 30 male	4 months	1. Weight loss 2. Comparison weight loss in those with metabolic syndrome	1. Decreased weight 2. No difference	5.9 kg decrease 2.5 kg/m^2^ BMI decrease

### Adult literature review

Ten adult studies (18 years and older) were also included (Table 2). There were 8 clinical trials and 2 review articles. Eight of the studies focused on individual weight loss and showed decreased weight. In addition, 5 studies reported improved biochemical parameters, 3 decreased rates of diseases associated with obesity (i.e., hypertension, dyslipidemia), 1 improved mental health, 1 increased fitness, and 2 improved quality of life.

**Table 2:** Literature review and study characteristics for adult studies.

Article	Date	Country	Study Design	Intervention	Population	Length	Primary Outcome (s)	Result	Weight Loss
Boden et al. (44)	2005	USA	Controlled Clinical Trial	Hospitalization and implementation of low-carbohydrate diet	10 individuals - Ages 46–64 - 7 female, 3 male *Exclusion: No type 2 diabetes*	2 weeks	1. Weight loss 2. Low carbohydrate diet effect on appetite, glucose, insulin	1. Decreased Weight 2. Decreased mean energy intake, hgb A1c, glucose, insulin sensitivity, triglycerides, cholesterol	1.65 kg decrease
Wiltink et al. (45)	2007	Germany	Randomized Controlled Trial	Inpatient behavioral or psychodynamic treatment	267 individuals - Ages 20–64 - 227 female, 40 male	7 weeks	1. Weight loss 2. Effect of behavioral vs. psychodynamic treatment	1. Decreased weight in 1/3 patients 2. Cognitive control and current physical activity predicted weight loss maintenance	3 kg decrease
Huerta et al. (20)	2010	USA	Controlled Clinical Trial	Inpatient hospitalization with low calorie liquid diet and exercise program (prior to surgery)	5 individuals - Ages: average 54.7 - 0 female, 5 male *Exclusion: not follow diet*	8–15 weeks	1. Weight loss 2. Effect of low calorie diet	1. Decreased weight 2. Decreased hgbA1c, hypertension, osteoarthritis, dyslipidemia	12.7 kg/m^2^ BMI decrease 38.86 kg decrease
Danielsen et al. (46)	2013	Norway	Controlled Clinical Trial	Inpatient lifestyle modification program with intensive physical activity, behavior modification sessions, dietary changes	77 individuals *33 controls* - Ages: 18–65 - 42 female, 29 male *Exclusion: pregnancy, unable walk 20 min*	10–14 weeks	1. Weight loss 2. Changes in body composition, cardiovascular risk, eating behaviors	1. Decreased weight 2. Decreased fat mass, blood pressure, glucose, cholesterol	17 kg decrease *10–14 weeks* 23.9 kg decrease *6 months* 20.3 kg *12 months*
Danielsen et al. (47)	2014	Norway	Controlled Clinical Trial	Inpatient lifestyle modification with physical activity, dietary changes	67 individuals *33 controls* - Ages 18–65 - 43 female, 24 male	10–14 weeks	1. Changes in life quality	1. Improved 2. Improved binge eating, depression, mental and physical health 3. Greater weight loss with longer inpatient interventions	Unspecified decrease
Wachsberg et al. (11)	2014	USA	Randomized Controlled Trial	Inpatient weight loss intervention with postdischarge phone follow-up	176 individuals - Ages 18–65yrs - 118 female, 58 male *Excluded: comorbidities, medications, depression, non- English speaking*	Varied, 6 month follow up	1. Weight loss 2. Weight maintenance	1. Decreased weight – *did not meet goal of >5% weight loss* 2. No significant difference between groups at 6 months	1.08 kg decrease *Vs. 1.35 kg decrease control*
Giordano et al. (48)	2017	Italy	Clinical Trial	Multidisciplinary program with nutritional and physical rehabilitation with psychological and educational intervention	146 individuals - Ages 19–79 - 83 female, 53 male	3 weeks	1. Weight loss 2. Reduction waist/neck circumference, glycemic control, blood pressure, cholesterol 3. Change in quality of life, physical performance	1. Decreased weight 2. Reduced circumference, 68% with glycemic control, improved cholesterol and blood pressure 3. Improved quality of life	3.9% BMI decrease
Lv et al. (2)	2017	USA	Review	Behavioral lifestyle interventions	1,862 individuals *12 studies* - Ages 30–54	Varied	1. Weight loss	1. 18/25 interventions in 12 studies reported clinically significant weight loss 2. Inpatient stays with high percentage weight loss	32–97% achieved 5% weight loss
Weinreich et al. (49)	2017	Germany	Clinical Trial	Inpatient rehabilitation with nutritional, behavioral, and exercise therapy modules	52 individuals -Ages 22–66 - 12 female, 40 male *Exclusion: Physical limitations, secondary obesity, history of bariatric surgery*	4 weeks	1. Weight loss	1. Decreased weight 2. Improved glucose, hgbA1c, blood pressure, body fat 3. Improved quality of life	7.1 kg 2.3 kg/m^2^
Rees et al. (8)	2021	Australia	Review	Varied, inpatient non-surgical interventions	1,146 individuals *12 articles, 10 studies* - Ages 18 and older - Amount female/male unspecified - Inclusion: adults, non-surgical weight loss intervention, Class II or III obesity, inpatient 1990–2019	0–17 weeks	1. Current weight loss research in inpatient setting 2. Comparison of different weight loss strategies	1. Gaps in research in non-surgical, holistic weight loss interventions 2. Statistically significant improvements in health inpatient vs. outpatient treatment 3. Variable approaches in countries considering obesity disease vs. lifestyle condition	Unspecified

### Patient cases

**Figure 2:**
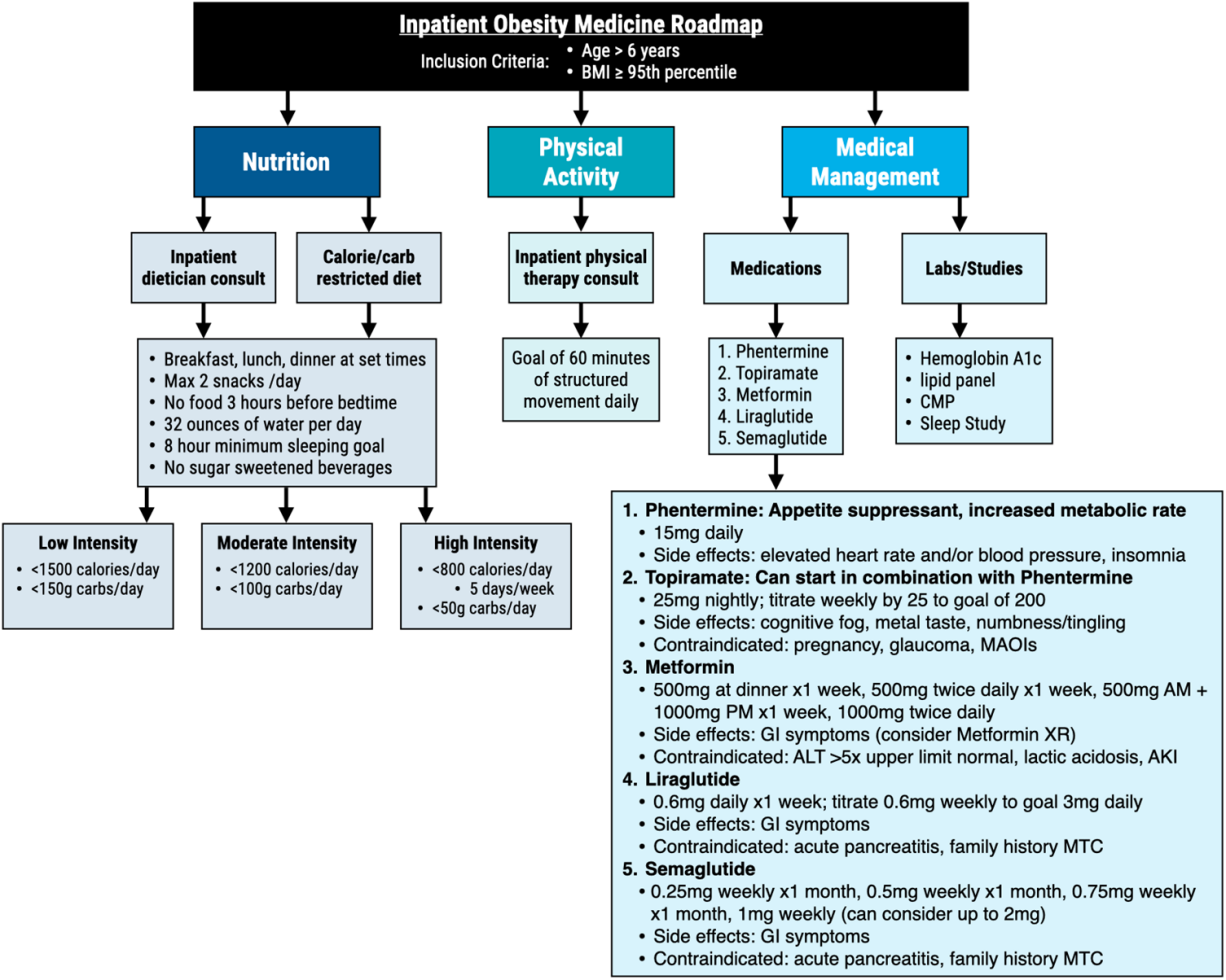
Model of inpatient obesity treatment roadmap with collaboration of obesity medicine specialists (MD/NP), nutritionists, nurses, and physical therapists to create individualized nutrition, activity, and medical plans for pediatric patients. BMI, body mass index; CMP, comprehensive metabolic panel; MAOI, monoamine oxidase inhibitors; XR, extended release; AKI, acute kidney injury; MTC, medullary thyroid carcinoma; GI, gastrointestinal.

We present three cases of youth with severe obesity who were hospitalized for acute medical problems while we simultaneously addressed weight-management. The severity of obesity in these youth was either impacting their present health or ability to undergo certain medical treatments. All three patients were treated following the inpatient obesity treatment roadmap developed at our institution that includes working with nutritionists, obesity medicine specialists (MD/NP), bedside nurses, and physical therapy to create an individualized nutrition, exercise, and anti-obesity medication plan for each patient (Figure 2).

### Case 1

**Figure 3:**
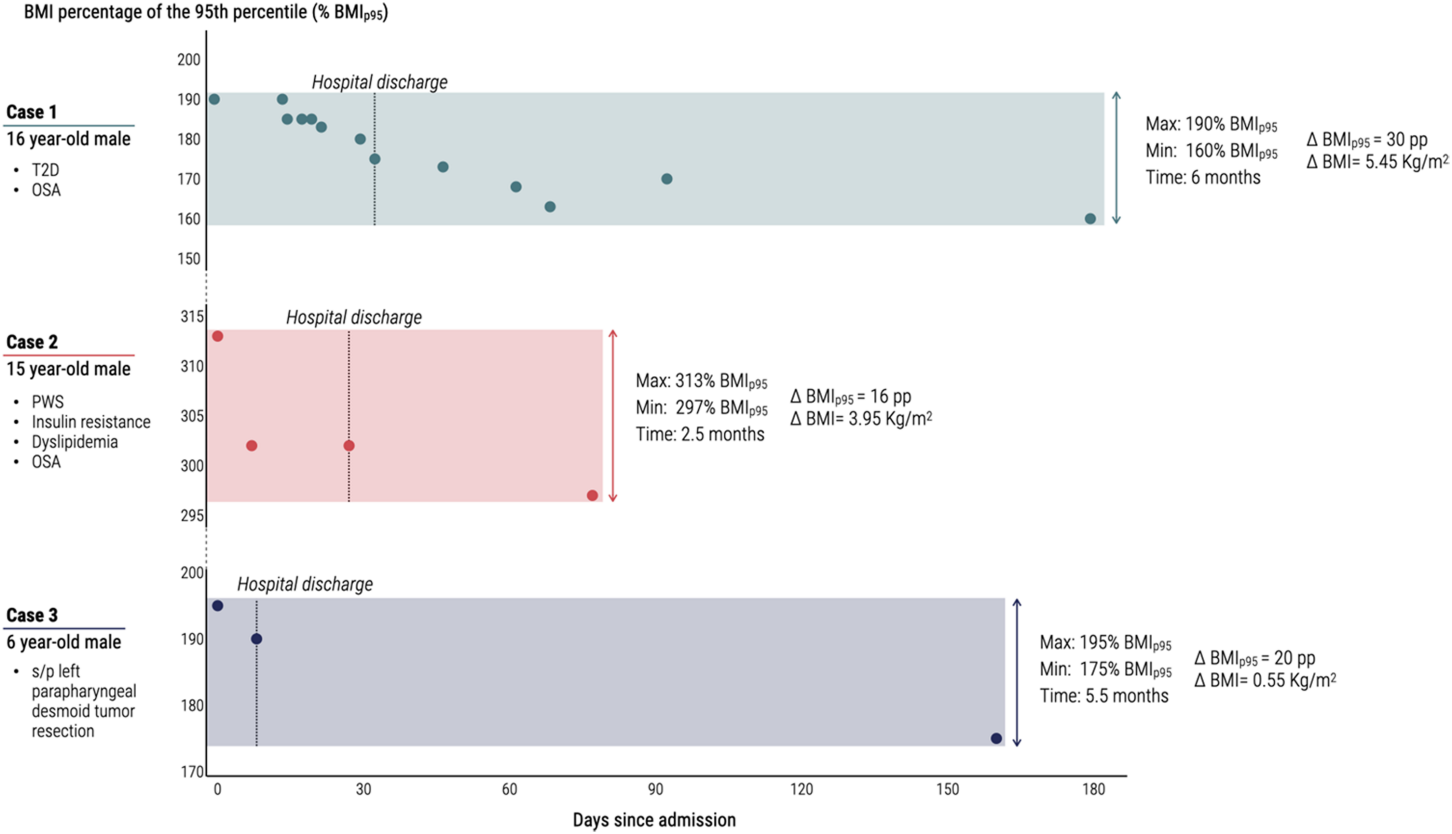
Change in BMI percent of the 95th percentile over time for three pediatric cases of inpatient obesity medical management. T2D, type 2 diabetes; OSA, Obstructive Sleep Apnea; PWS, Prader-Willi Syndrome; BMI, body mass index; Kg/m^2^, kilograms per meter squared; % BMI_p95_, BMI percentage of the 95th percentile; pp, percentage points; *Δ*BMI_p95_, change in BMI percentage of the 95th percentile; *Δ*BMI, change in absolute BMI.

Case 1 is a 16-year-old male who presented with well-controlled type 2 diabetes, obstructive sleep apnea (OSA), severe obesity, and acute onset severe back pain with limited mobility. Initial biochemical evaluation was concerning for acute lymphocytic leukemia (ALL), requiring magnetic resonance imaging (MRI) to determine staging and appropriate treatment. However, due to limitations of the MRI machine at the pediatric center, the patient was unable to obtain imaging secondary to body habitus presenting a significant diagnostic challenge. The obesity medicine team was consulted for assistance with acute weight loss to promote ability for accurate diagnosis, prognosis, and initiation of an appropriate treatment regimen for his oncologic process. The patient was admitted to inpatient rehabilitation for two weeks. On admission, his type 2 diabetes was controlled with insulin and metformin and he had a hgA1c of 6.4. However, due to worsening hepatotoxicity, his Metformin was discontinued. Given his new onset AL.L and concern for chemotherapy induced pancreatitis, the oncology team did not feel comfortable starting a glucagon-like peptide 1 (GLP-1) medication. Interventions included placing the patient on the institutional inpatient obesity protocol including: (1) intake with a registered dietitian; (2) dietary composition: 1,500 calorie per day, less than 150 grams of carbohydrates, no sugar-sweetened beverages, 35 grams of fiber, 32 ounces of water; (3) structured mealtimes (3 meals and 2 snacks); (4) daily physical therapy as tolerated; (5) initiation of weight loss medications (phentermine 15 mg daily). One week after initiation of this protocol, his weight was down trending and he was tolerating phentermine without side effect. Given lack of side effects but increasing hunger with goal of continued weight loss in efforts to obtain an MRI, his phentermine was increased to 37.5 mg daily and dietary program intensified to 1,200 calories and less than 100 grams of carbohydrates daily. At admission, his weight was at 190% BMI_p95_ (Figure 3). Upon discharge and after two weeks after initiation of his individualized weight-management plan, it decreased to 175%BMI_p95_ and his hgba1c improved to 6.0. He also reported decreased appetite and no side effects throughout his treatment. He continued his outpatient weight-management program with monthly virtual visits with obesity medicine specialists and dietitian. One month after discharge, his %BMI_95_ had decreased to 165% BMI_p95_, and by month six, to 160% BMI_p95_ (associated with a total BMI reduction of 4.85 kg/m^2^ and BMI *z*-score reduction of 0.12 standard deviations).

### Case 2

Case 2 is a 15-year-old male with Prader-Willi Syndrome, class III obesity, impaired fasting glucose, hypertriglyceridemia, and obstructive sleep apnea (OSA) controlled on continuous positive air pressure (CPAP) admitted for acute respiratory failure and placed on a ventilator. Patient had a history of excessive weight gain due to hyperphagia associated with significant anxiety exacerbated by the Covid-19 pandemic. Outpatient, he was taking Metformin 1,000 mg twice daily and Topiramate 100 mg nightly for off-label weight control. Family had completed three, intensive outpatient lifestyle modifications programs and set up a food safe zone (locked cabinets and food storage units) with limited success. Upon admission, he had a 40-pound gain since his last outpatient encounter four months prior and his respiratory function had worsened significantly in response to this weight gain with blood gases demonstrating carbon dioxide retention levels in the upper 70s. He underwent extensive evaluation for hypertension, cardiac dysfunction, and worsening obstructive sleep apnea and his acutely worsened respiratory status was thought to be largely secondarily to his weight gain. This evaluation included a normal electrocardiogram, echocardiogram and renal ultrasound. He underwent a polysomnography which was abnormal due to breath stacking, hyperventilation, and hypoxemia for which his CPAP machine was adjusted. He also had a number of screening labs during his hospitalization with normal thyroid function (TSH 2.62 uIU/ml, T4 7.5 mcg/dl), free cortisol (0.18), ACTH (21) hgbA1c (5.3%), lipid panel (triglycerides 94, total cholesterol 150). Notably he had low testosterone (Free 2.09 ng/dl, total 83 ng/dl), LH (0.31 mIU/mL), FSH (<0.66 mIU/ml), IGF-1(26 ng/ml) and IGF-BP3 and (0.8 mg/L) ultimately started on 100 mg Testosterone supplementation.

The obesity medicine team was consulted by pulmonology to address diagnostic and treatment challenges by supporting acute weight loss interventions while inpatient. This was in an effort to optimize respiratory control for ability to discharge home. The personalized intervention for this patient included placing him on the institutional inpatient obesity protocol including: (1) intake with a registered dietitian; (2) dietary composition: 800 calories daily, less than 90 grams of carbohydrates, no sugar-sweetened beverages, 35 grams of fiber, 32 ounces of water; (3) structured mealtimes (2 meals and 2 snacks); (4) daily physical therapy as tolerated; (5) optimization of weight loss medication regimen (topiramate increased to 200 mg nightly and initiation of semaglutide 0.25 mg weekly). He remained admitted to the rehabilitation unit for one month, during which, his %BMI_p95_ decreased from 313% to 300% BMI_p95_ (Figure 3). At the same time, his ventilatory needs decreased with successful transition to continuous positive airway pressure (CPAP) overnight and oxygen by nasal cannula during the day, allowing for safe discharge home. He reported no side effects from the medications during this time period. Given lack of side effects and meeting goal of decreasing respiratory support, no further medication changes were made inpatient. Upon discharge, he was enrolled in the outpatient weight-management program and attended monthly visits with obesity medicine specialists and dietitian. Two and a half months after discharge, his weight had decreased to 297% BMI_p95_ (associated with a total BMI reduction of 1.2 kg/m^2^ and BMI *z*-score reduction of 0.01 standard deviations). His triglycerides and hgba1c were stable at 94 and 5.3% respectively at this time and Testosterone increased with injections.

### Case 3

Case 3 is a 6-year-old male with history of a parapharyngeal desmoid tumor resected in June 2019, who had subsequent weight gain of 20 kg over the next two years in association with the Covid-19 pandemic. He was getting serial MRIs with sedation for tumor monitoring, which required admission for respiratory support in the context of his obesity. The obesity medicine team was consulted by oncology and anesthesiology to support acute weight loss interventions while inpatient in an effort to minimize airway risk with his recurrent sedation needs. He was placed on the institutional inpatient obesity protocol including: (1) intake with a registered dietitian; (2) dietary composition: 1,200 calories daily, less than 100 grams of carbohydrates, no sugar-sweetened beverages, 35 grams of fiber, 32 ounces of water; (3) structured mealtimes (3 meals and 2 snacks); and (4) thirty minutes of moderate intensity physical activity daily. One challenge the obesity medicine team faced during the goal of acute weight loss was parental preference not to start anti-obesity medications. He did continue to have normal Hgb A1c (5.1–5.2%) as well as normal lipid panel (triglycerides 74, total cholesterol 161) but elevated alanine aminotransferase and aspartate aminotransferase of 62 and 48 respectively. He remained admitted to the rehabilitation unit for one week with associated weight stabilization. Upon discharge, he was enrolled in the outpatient weight-management program and met with obesity medicine specialists and a dietitian monthly. Five months after discharge, his weight had decreased from 195% BMI_p95_ to 175% BMI_p95_ (Figure 3).

## Discussion

Pediatric obesity affects acute, inpatient management of non-weight related conditions by impeding efficiency of diagnostic work-ups, delaying diagnosis, limiting efficacy of medications, and contributing to longer hospital stays and higher rates of complications (4, 12, 13, 50–54). Despite severe obesity directly impacting patient care, safety, and costs, there is minimal guidance on when or how to intervene for weight-management in the inpatient settings. Overall, studies reveal that nutrition and weight-management protocols or universal counseling are limited while inpatient, even though inpatient treatments are typically most effective, which suggest there is a need and an opportunity to fill this treatment/intervention gap in research and clinical practice (10, 22, 23).

This review highlights the paucity of data available on inpatient weight-management interventions. Two categories of studies came out of this investigation: (1) inpatient admission for the purpose of weight-management (75% of studies included) and (2) weight-management as a component of an inpatient admission for another acute condition. Consistently, studies that compare inpatient vs. outpatient weight-management interventions show greater weight loss in the inpatient arm of the interventions. It is well-reported in the literature that controlled nutrition trials often have greatest success for weight-management across the lifespan, however there are challenges in incorporating those treatment strategies into real-world settings. Despite successes of inpatient weight-management interventions, the labor and cost required to implement them are significant and the disruption to an individual's daily schedule often prevents them from being enacted on a larger scale. However, even when patients are admitted for treatment of other conditions, we can learn from these studies on how to best harness successful treatment strategies and utilize hospitalization time, which creates an opportunity to start weight-management conversations and care (26, 29, 44).

With growing rates of obesity in youth, there is a pressing need to understand how to optimally address obesity within the inpatient setting. Our single center experiences highlight examples of how these needs arise and how there are rarely clear guidelines on how to consider weight-management during hospitalizations. This gap led our team to create a roadmap for addressing pediatric obesity within inpatient settings. Given growing number of multi-disciplinary, pediatric outpatient weight-management programs, there exists an opportunity to learn from these outpatient practices and design protocols supporting youth with obesity, regardless of health system setting– outpatient or inpatient. At our center, this team consists of the following: endocrinology, obesity medicine, nursing, hospital medicine, physical therapy, dietitians, and rehab specialists. To create our protocol, we referenced current clinical practice guidelines for pediatric obesity medicine, and incorporated weight loss medications, prescriptive nutrition, and activity plans focused around a structured-day approach. Each plan is individualized to the youth's medical needs and acute weight loss goals, as determined by primary team. It also incorporates transition plans from inpatient to outpatient settings. Longitudinal controlled trials are required to further investigate how inpatient protocols can be utilized and to determine the most appropriate evidenced-based strategy to support both acute weight loss and maintenance over time (55, 56).

Our case series show that multi-disciplinary execution of an inpatient obesity protocol can result in BMI reduction and support timely diagnostic work-up, safe treatment, and improved outcomes in the short term. Importantly, long term follow up is necessary to ensure continued weight loss or maintenance. All three of our cases demonstrated acute weight-loss allowing for decreased respiratory support needs and/or the ability to obtain important diagnostic imaging. No significant side effects were reported by the individuals. The adolescent patients (Case 1 and Case 2) described gratefulness for the weight loss interventions because it presented them opportunities to take control of an important aspect of their health. The patient in Case 1 reported feeling a lack of control with their new diagnosis of cancer and a relief with this achievable focus of weight loss. The parents of the patient in Case 3 reported appreciation for treating obesity as a medical disease and reducing stigma. Given the hope for longevity with these weight loss interventions, it was important to our team that the patients also felt empowered and had a realistic plan for home after their inpatient hospitalizations.

### Limitations

Naturally, this review is not without limitations. First, by including “inpatient,” “obesity,” and “intervention” as our search terms, we may have excluded studies using synonyms. We only searched PubMed, so may have missed articles in other databases, but felt PubMed would be the most comprehensive source of literature relevant to inpatient pediatric obesity interventions. We also excluded non-English literature, while including literature from other countries. Second, the significant differences in methodology and reported data complicate any quantitative comparison across studies. Additionally, few studies report information on non-completers of the interventions or other potential confounding factors, so selection bias may have affected some outcomes. Limitations of our case series include only examining three patients of different ages with varied length of inpatient stays, comorbidities, and medications. Additionally, our patient follow up was confined to 6 months following hospital discharge and it is important to note that weight loss or weight maintenance requires long term follow-up and often individuals may gain weight back. Our focus was on weight loss in the acute setting to assist with specific medical needs including ability to obtain imaging or treat acute illness.

## Conclusions

The prevalence of pediatric obesity continues to rise. Obesity impacts acute inpatient management of pediatric conditions by resulting in delays in diagnosis, compromised treatment plans, prolonged lengths of stay, and higher complication rates, which all contribute to increased health care costs and worse outcomes. The limited studies addressing this topic demonstrate that weight-management can and should be considered in an inpatient setting, either as the primary outcome or as an adjunct to other medical treatment. Further investigation is required to design and implement inpatient weight-management protocols for youth with obesity while hospitalized.

## Data Availability

The original contributions presented in the study are included in the article/Supplementary Material, further inquiries can be directed to the corresponding author/s.
